# Technical and clinical outcomes of microwave ablation for HCC: a single-center retrospective analysis of percutaneous ultrasound-guided, intraoperative ultrasound-guided and CT hepatic arteriography-guided approaches

**DOI:** 10.1007/s11547-026-02207-y

**Published:** 2026-03-24

**Authors:** Riccardo Muglia, Piergiorgio Laudicina, Alessandro Barbaro, Francesco Saverio Carbone, Martina Bertuletti, Ludovico Dulcetta, Chiara Pavoni, Martijn Meijerink, Bruno Calazans Odisio, Robbert Puijk, Paolo Marra, Sandro Sironi

**Affiliations:** 1 Radiology Unit, ASST Papa Giovanni XXIII Hospital, Bergamo, Italy; 2 School of Medicine, University of Milano-Bicocca, Milan, Italy; 3 Hematology and Bone Marrow Transplant Unit, ASST Papa Giovanni XXIII Hospital, Bergamo, Italy; 4 Department of Radiology and Nuclear Medicine, Amsterdam University Medical Centers, Location VUmc, Amsterdam, The Netherlands; 5 Cancer Center Amsterdam, Research Program, Amsterdam, The Netherlands; 6 Department of Interventional Radiology, Division of Diagnostic Imaging, The University of Texas MD Anderson Cancer Center, Texas, USA; 7 OLVG Hospital, Amsterdam, The Netherlands; 8 Piazza Dell’Ateneo Nuovo 1, 20126 Milan, Italy; 9 Piazza OMS 1, 24129 Bergamo, Italy

**Keywords:** HCC, Microwave ablation, Ultrasound-guided ablation, Intraoperative ultrasound-guided ablation, CT hepatic arteriography-guided ablation

## Abstract

**Objectives:**

To compare technical/clinical outcomes of microwave ablations (MWA) for hepatocellular carcinoma (HCC) performed with percutaneous ultrasound (US)-guidance, intraoperative ultrasound (IOUS)-guidance or CT hepatic arteriography (CTHA)-guidance.

**Materials & methods:**

This single-center retrospective study included 111 non-randomized patients (M:F = 91:20, median age 66y, range 51–86) with 200 HCCs (BCLC 0-A-B), treated with 136 MWA procedures (66 US-guided, 36 IOUS-guided, 34 CTHA-guided) between July 1, 2017, and January 31, 2025, with at least 6 months of clinical and CT/MRI follow-up. We evaluated patients’ and nodules’ characteristics, radicality (absent residual tumor at follow-up), local tumor progression, additional treatments, adverse events (CIRSE classification) and mortality. For patients undergoing multiple ablations, clinical outcomes were analyzed in relation to the first treatment.

**Results:**

One nodule was ablated in 94/136 (69.1%) procedures, 2 nodules in 25/136 (18.4%), > 3 in 17/136 (12.5%). We encountered 13 adverse events, with the highest severity in IOUS-guided MWAs (1 grade 3, 2 grade 6).

Fifty-eight patients (52.3%) progressed in other segments, subsequent treatments were performed in 57/111 (51.3%) patients and 28/111 (25.2%) died during follow-up.

IOUS-guided MWA was associated with the highest radicality rate (56/60, 93.3%, *p* = 0.04) compared to CTHA-guided (45/51, 88.2%) and US-guided (72/89, 80.9%) ablations. Treating multiple nodules increased complication risk (*p* = 0.003), impacting on radicality (*p* = 0.032). No differences were found for overall survival (*p* = 0.07) or progression-free survival (*p* = 0.584) among the techniques.

**Conclusions:**

IOUS-guidance for HCC ablation provided a higher radicality rate compared to CTHA- and ultrasound-guidance techniques, but carried a higher risk of severe complications.

## Highlights

- Despite the variety of techniques employed, there is no consensus on which imaging guidance method best balances technical success, oncological efficacy and patient safety in the context of microwave ablation for HCC.
- IOUS-guided microwave ablation achieved the highest radicality rates, although with severe complications; US-guided ablation was effective for well-visualized lesions; CTHA-guided ablation offered an optimal balance between safety and efficacy.
- Our findings support a tailored approach to imaging guidance in HCC microwave ablations, considering lesion characteristics, patient condition and institutional resources to optimize outcomes.

## Introduction

Hepatocellular carcinoma (HCC) is the most common primary liver malignancy and a leading cause of cancer-related mortality worldwide. In patients with underlying liver cirrhosis, which represents the most common background condition for HCC, treatment strategies must balance oncological efficacy with preservation of liver function[1]. Among the available options, thermal ablation has emerged as a safe and effective treatment modality, offering outcomes comparable to surgical resection in selected patients, particularly those with early-stage disease and impaired hepatic reserve[2, 3].

Over the years, microwave ablation (MWA) has gained increasing popularity due to its ability to generate higher intratumoral temperatures, larger ablation volumes and shorter procedure times, making it suitable for treating larger and less accessible lesions[4]. These advantages have positioned MWA as a valid alternative to surgical resection in cirrhotic patients with HCC[5].

A critical component of a successful MWA is the accurate imaging guidance, which ensures precise needle placement and complete tumor coverage, while minimizing damage to liver parenchyma and adjacent structures[6, 7]. Various imaging techniques are available to guide ablation procedures, including percutaneous ultrasound (US), intraoperative ultrasound (IOUS) and computed tomography hepatic arteriography (CTHA)[8–10]. Each modality presents specific advantages and limitations regarding lesion detectability, spatial resolution, procedural invasiveness and operator dependency[11].

The choice of real-time imaging guidance techniques is often influenced by the expertise and technological availability within each center. Percutaneous US is widely accessible and allows real-time guidance, but may be limited by suboptimal visualization of deeply located or isoechoic lesions[12]; IOUS provides enhanced lesion detectability and facilitates immediate treatment during surgery, but exposes the patients to the risks of an invasive intervention, while CTHA offers superior contrast enhancement and sensitivity for small or inconspicuous tumors, although it is more invasive than percutaneous US-guidance and technically demanding[13, 14].

Despite the variety of techniques employed, there is currently no consensus on which imaging guidance method best balances technical success, oncological efficacy and patient safety in the context of MWA for HCC. The aim of our study was to evaluate our institutional experience in HCC MWA in cirrhotic subset, comparing three different imaging guidance strategies: US-guided, IOUS-guided and CTHA-guided approaches.

## Material and methods

### Study design and patient selection

This retrospective analysis was performed at a single tertiary referral center (-blinded for review-), adhering to the ethical standards of the Declaration of Helsinki; specific approval from the Institutional Review Board was obtained (protocol n° 0040510/25). All participants provided informed consent for anonymized data usage and publication.

All patients included in this study were selected by a multidisciplinary tumor board dedicated to liver malignancies, comprising specialists in hepatology, surgery (general and transplant), interventional radiology, oncology, radiation therapy and nuclear medicine, following the BCLC guidelines[15].

As per Institutional protocol, every patient candidate for MWA was screened by an ambulatorial US no more than one month prior to the treatment: whether the liver lesion was visible and accessible, and MWA was judged clinically appropriate, a US-guided treatment was proposed. Differently, if the lesion was deeply located or not conspicuous, an IOUS-guided ablation or a CTHA-guided ablation was prescribed, based on the patient’s clinical conditions, nodule position and liver vascular anatomy. For these reasons, the distribution of the image-guidance techniques described in this study was non-randomized.

Our primary endpoint was to compare technical outcomes of MWA for HCC performed with US-guidance, IOUS-guidance and CTHA-guidance, between July 1, 2017, and January 31, 2025; in particular, we intended to evaluate the radicality rate, local tumor control and adverse events attained with each imaging technique. As a secondary endpoint, we accounted for the evaluation of clinical outcomes related to patients undergoing MWA with these three image-guidance techniques, especially in terms of local tumor progression and survival.

To be eligible for inclusion, patients had to present at least one HCC confirmed either through Liver Imaging Reporting and Data System (LI-RADS) criteria or histologically via biopsy[16]. Only patients with BCLC stage 0 or A were routinely included; BCLC stage B patients were considered only if thermal ablation was part of a liver transplant downstaging protocol. Moreover, all included patients had a minimum follow-up of 6 months, including a clinical examination and a contrast-enhanced CT/MRI. Alternatively, patients were excluded if < 18 years old, had anesthesiological contraindications, underwent previous treatments on the target HCC, were ablated with other technologies than microwave or had incomplete clinical or radiological data. For those patients receiving more than one MWA, only the first procedure was analyzed for the clinical endpoints of this study; for the evaluation of technical outcomes, every procedure was analyzed singularly.

Following the inclusion/exclusion criteria, we analyzed clinical outcomes for 111 patients (M:F = 91:20, median age 66 years, range 51–86; 54 US-guided, 35 IOUS-guided, 22 CTHA-guided MWAs), and technical outcomes for 136 MWAs (including repeated ablations in 25 patients; 66 US-guided, 36 IOUS-guided, 34 CTHA-guided), aiming to treat 200 HCCs. Different guidance techniques were never combined in the same procedure.

Data were extracted from a prospectively collected institutional registry, which included detailed clinical, imaging and procedural information for all patients. The dataset analyzed included demographic variables, etiology of liver disease, BCLC classification, lesion characteristics (size and location), procedural details, adverse events (categorized according to the CIRSE Classification System for Complications[17]), length of hospital stay, operative approach (open surgery or videolaparoscopy, for IOUS-guided ablations only), radiation exposure (for CTHA-guided ablations only), ablation radicality, tumor persistence, local tumor progression, local tumor control, following treatments and survival.

Radical ablation was defined as the absence of residual contrast enhancement adjacent to the treated area on post-procedural imaging, while tumor persistence as the presence of an enhancing nodule adjacent to the necrotic volume at the first imaging control. Local tumor progression, instead, was defined as the growth of new nodules of HCC during follow-up, after at least one contrast-enhanced CT/MRI documenting no residual viable tumor at the ablative margins. Then, with local tumor control, we intended the eradication of the target nodule with one or more treatments[18].

When a nodule sized ≤ 30 mm at the preoperative evaluation grew to > 30 mm at the time of MWA, the intervention was performed with at least one antenna repositioning to cover the full nodule volume[19]; when ≤ 3 HCCs were visible at the preoperative evaluation, but ≥ 1 new nodule with typical HCC characteristics was discovered at the moment of the intervention, synchronous ablation was performed in the same procedure.

All patients undergoing MWA were prescribed a clinical and radiological follow-up (with contrast-enhanced CT or MRI) at 1 month, then every 3 months for the first year and every 6 months from the second year on. All preoperative and post-operative imaging was reviewed by both a senior and a junior radiologist (-initials blind for review-), in a blind fashion to clinical and technical data.

### US-guided ablation

Percutaneous MWA under US-guidance was commonly performed with local anesthesia and moderate sedation, tailored to the patient’s condition and tumor location[20]. After sterile preparation, an initial ultrasound scan identified the hepatic lesion, evaluating its size, shape and relationship to critical structures such as bile ducts, vessels and neighboring organs.

**Fig. 1:**
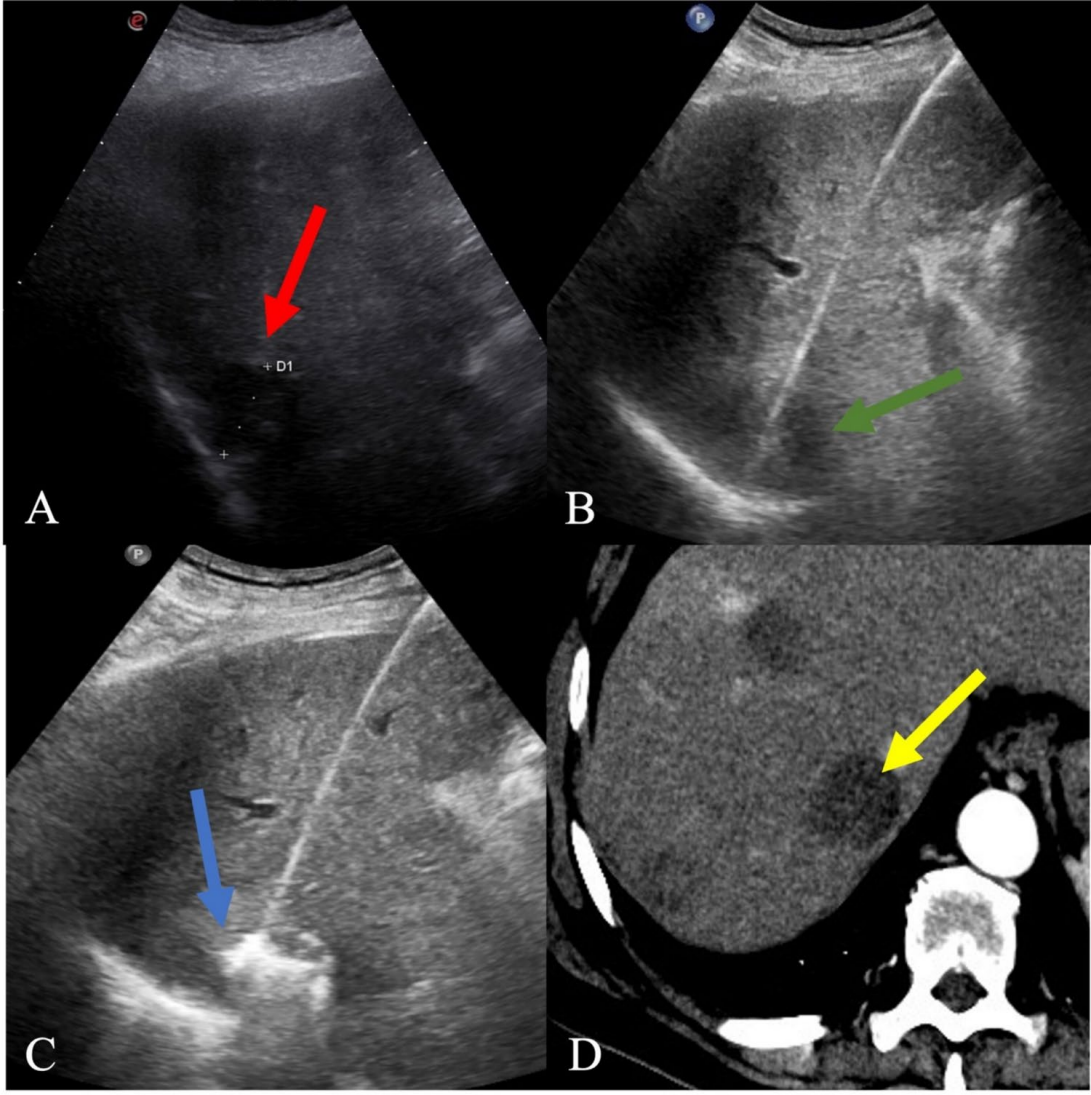
**A** Hypoechoic HCC (red arrow) of 27 mm in segment VIII, evaluated for percutaneous, US-guided ablation; **B** Transhepatic microwave antenna (green arrow) with the tip inside the nodule; **C** Hyperechoic transformation of the nodule (blue arrow) after 8 min of 100W ablation, due to gas bubbles; **D** 6 months post-operative contrast-enhanced CT scan, demonstrating ablation radicality around necrotic volume (yellow arrow)

Using real-time US-guidance, a microwave antenna (typically 15–20 cm, Emprint, Medtronic, USA) was inserted through the skin and directed toward the tumor, using a freehand technique. Precise trajectory planning avoided damage to non-target tissues. Once the antenna was advanced in the tumor’s core, placement was verified in multiple planes with B-mode imaging. During the procedure, hyperechoic microbubbles appeared on US as an indicator of effective tissue heating [Fig. 1: A) Hypoechoic HCC (red arrow) of 27 mm in segment VIII, evaluated for percutaneous, US-guided ablation; B) Transhepatic microwave antenna (green arrow) with the tip inside the nodule; C) Hyperechoic transformation of the nodule (blue arrow) after 8 min of 100W ablation, due to gas bubbles; D) 6 months post-operative contrast-enhanced CT scan, demonstrating ablation radicality around necrotic volume (yellow arrow)].

Finally, the ablation zone was evaluated by contrast-enhanced US and checked for immediate complications[21]. Patients were monitored post-procedure and typically discharged after one or two night's observation, depending on clinical status.

### IOUS-guided ablation

Performed under general anesthesia, IOUS-guided ablation could be performed through laparoscopic or open surgical approaches. The choice between laparoscopic and open surgery was based on multiple factors, including tumor location, patient comorbidities, previous abdominal surgeries and eventual transplant program, with minimally invasive approaches preferred when oncologically and technically feasible.

In laparoscopic procedures, abdominal access was obtained via a 12-mm optical trocar, with pneumoperitoneum established at 12–14 mmHg. A second trocar was placed depending on tumor location[22]. A high-frequency linear ultrasound probe was introduced through a trocar to perform intraoperative liver scanning. Additional trocars might be used to improve lesion access through adhesiolysis[23]. Once the tumor was visualized, a 30-cm antenna was percutaneously advanced into the lesion under coaxial laparoscopic and ultrasound guidance.

For open surgery, access was gained via a subcostal incision, and a micro-convex US probe was used to locate the lesion. A 15–20 cm microwave antenna was then inserted under direct US-guidance. Ablation started at 100W, with time adjustment based on lesion size and device-specific protocols[24].

### CTHA-guided ablation

CTHA-guided ablation was conducted under general anesthesia. After a 5-Fr transfemoral arterial access, we catheterized the proper hepatic artery with a 5-Fr Cobra C2 or Simmons 1 (Tempo Aqua, Cordis, USA). Correct positioning was confirmed with iodinated contrast injection, and the catheter was secured to minimize displacement risk.

For pretreatment planning, a CTHA was acquired by infusing a 1:1 saline-contrast mixture at 4 mL/s, with arterial and portal venous phases imaged at 6 and 22s post-injection[25]. Then, CT-fluoroscopy guided antenna insertion into the tumor, supported by intermittent small-volume contrast injections to enhance visualization.

**Fig. 2:**
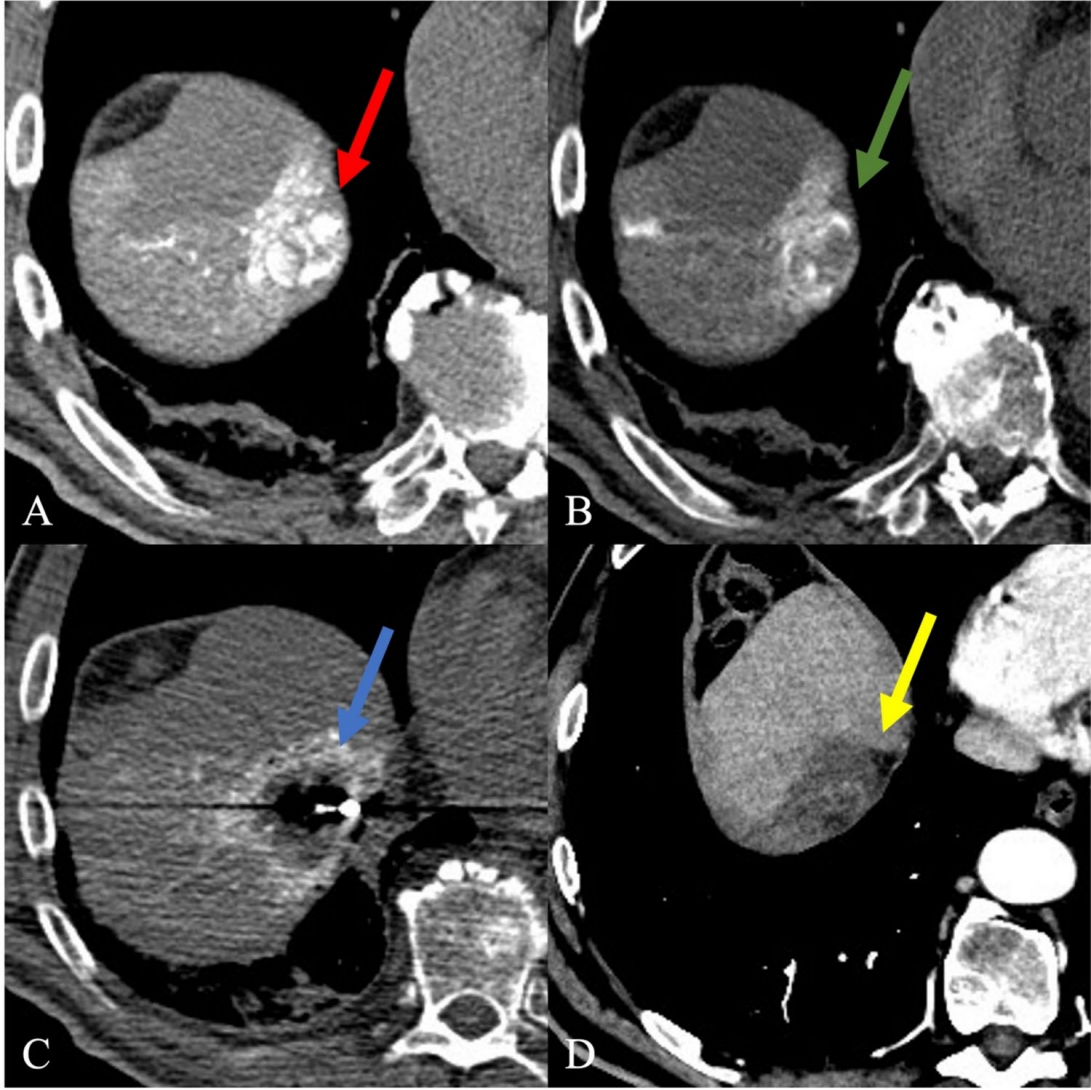
**A** Twenty-five mm HCC nodule in segment VII with contrast enhancement at arterial phase CTHA (red arrow) and **B** its enhancing capsule at venous phase (green arrow); **C** after deploying the tip of microwave antenna inside the nodule (blue arrow), an ablation of 7 min at 100W was performed; **D** after one year from the ablation, the absence of contrast enhancement around the ablation volume confirms the ablation radicality (yellow arrow)

After nodule ablation, a second CTHA was acquired to confirm technical success and detect eventual complications [Fig. 2: (A) Twenty-five mm HCC nodule in segment VII with contrast enhancement at arterial phase CTHA (red arrow) and (B) its enhancing capsule at venous phase (green arrow); (C) after deploying the tip of microwave antenna inside the nodule (blue arrow), an ablation of 7 min at 100W was performed; (D) after one year from the ablation, the absence of contrast enhancement around the ablation volume confirms the ablation radicality (yellow arrow)]. The catheter and sheath could be then removed, and femoral hemostasis was achieved by manual compression.

### Statistical analysis

Descriptive statistics were used to summarize patient characteristics at baseline. Categorical variables were reported as numbers and percentages, whereas continuous variables were summarized using medians and ranges.

Patients were stratified based on their survival status (alive/deceased) at the last follow-up. The Chi-square test (or Fisher’s exact test, as appropriate) was used to evaluate differences between groups for categorical variables, while the t-test or Wilcoxon–Mann–Whitney test was applied for continuous variables, depending on the distribution.

The association between imaging guidance and technical and clinical outcomes was assessed using the Wilcoxon–Mann–Whitney test for continuous dose data and Chi-square test for dichotomized dose data, with the optimal cut-off identified by Receiver Operating Characteristic (ROC) curve analysis.

Univariate and multivariable logistic regression models were used to identify the predictors of complete radiological response. Stepwise regression was used to select the best predictors for the multivariate model. Odds ratios (OR) and 95% confidence intervals (CI) were calculated.

Survival was estimated using the Kaplan–Meier method. Univariate and multivariable Cox proportional hazards regression models were used to identify predictors of mortality, and stepwise regression was employed to select the best predictors for the multivariable model. Hazard ratios (HR) and 95% confidence intervals (CIs) were reported.

For all the analyses, two-tailed p-values < 0.05 were considered statistically significant. Statistical analyses were performed using STATA software, version 16.1 (StataCorp LP, College Station, USA).

## Results

### Clinical and technical features

Among the 111 patients included, BCLC stages were distributed as follows: 33 patients (29.7%) were classified as BCLC 0, 62 (55.9%) as BCLC A and 16 (14.4%) as BCLC B.

Of the 200 HCC tumors treated, 89 (44.5%) underwent US-guided ablation, 60 (30%) IOUS-guided ablation and 51 (25.5%) CTHA-guided ablation.

A single lesion was targeted in 94/136 procedures (69.1%), two lesions in 25/136 procedures (18.4%) and more than three in 17/136 procedures (12.5%). Lesions were distributed across multiple liver segments, with segment VIII being the most frequent location in all three groups:, 23/89 (25.8%) in the US-guided group, 20/60 (33.3%) in the IOUS group and 21/51 (41.2%) in the CTHA group. Median lesion sizes were comparable across groups: 19 mm (range 7–53 mm) for US-guided, 16 mm (range 6–50 mm) for IOUS-guided and 17 mm (range 7–41 mm) for CTHA-guided procedures (*p* = 0.05).

Within the IOUS-guided cohort, 24/36 procedures (66.6%) were laparoscopic and 12/36 (33.3%) were performed via open surgery. The median radiation dose delivered during CTHA-guided ablations was 3281 mGycm2 (range 983–13.797 mGy cm^2^).

We repositioned the antenna due to interval tumor growth in 7/51 CTHA-guided cases (13.7%) and in 15/89 US-guided cases (16.9%).

Median hospital stay was 4 days across all groups, with ranges of 1–20 days for US-guided MWA, 1–51 days for IOUS-guided and 1–13 days for CTHA-guided procedures.

The median follow-up was 28 months (range 6–90), with differences across subgroups: 31 months (13–46) for US-guided, 46 months (20–90) for IOUS-guided and 15 months (6–26) for CTHA-guided patients.

The summary of population variables is resumed in Table 1 (demographics, nodules’ characteristics and principal clinical variables).

**Table 1:** Demographics, nodules’ characteristics and principal clinical variables

Population summary				
Variable	Total	CTHA-guided	IOUS-guided	US-guided
Number of patients	111	22	35	54
Gender (M:F)	91:20	18:4	29:6	44:10
Median age (range)	66 (51–86)	67 (51–83)	66 (52–83)	66 (51–86)
MWA procedures	136	34	36	66
HCCs treated	200	51	60	89
BCLC stage				
Stage 0	33	6	11	16
Stage A	62	13	20	29
Stage B	16	3	4	9
Lesions per procedure				
1 lesion	94	25	18	49
2 lesions	25	4	13	12
≥ 3 lesions	17	5	5	5
Median HCC size	17 (7–53)	17 (7–41)	16 (6–50)	19 (7–53)
Adverse events	13	6	3	4
Grade ≥ 3 adverse events	4	0	3	1
Deaths	28	1	13	14
Radical ablation	173	45	56	72
Progression free survival	11 (1–90)	9 (1–26)	26 (1–90)	9 (1–41)
Overall survival	24 (0–81)	15 (6–26)	34 (0–81)	27 (3–34)
Median follow-up	28 (6–90)	15 (6–26)	46 (20–90)	31 (13–46)

### Factors affecting outcomes

Radicality was achieved in 173/200 tumors (86.5%): 72/89 (80.9%) in the US-guided group, 56/60 (93.3%) in the IOUS-guided group and 45/51 (88.2%) in the CTHA-guided group. For those 27/200 (13.5%) persistent HCCs after ablation, local tumor control was attempted by repeat ablations (17/27 patients, 63.0%), transarterial chemoembolizations (TACE, 5/27 patients, 18.5%), transarterial radioembolizations (TARE, 3/27 patients, 11.1%), orthotopic liver transplant (OLT, 1/27 patient, 3.7%) and systemic therapy (1 patient). Apart from the only patient undergoing systemic therapy, local tumor control was reached in almost all cases (199/200, 99.5%).

Across the 136 ablation sessions, 13 adverse events (9.6%) were recorded (Table 2): 4 events (30.8%) occurred after US-guided MWA, 3 (23.1%) after IOUS-guided MWA and 6 (46.2%) after CTHA-guided MWA. Of the 4 US-guided complications, three were grade 2 (two arterioportal fistulas and one hemoperitoneum) and one was grade 3 (a biliary-colic fistula). IOUS-related complications included one grade 3 event (a biliary fistula requiring percutaneous embolization) and two grade 6 events: after an intestinal perforation occurring during adhesiolysis, one patient underwent repeat surgery to attempt a resection, but ultimately developed a fatal multiorgan failure; the other event was related to uncontrollable capsular bleeding, which could not be arrested despite both endovascular and surgical interventions. All CTHA-related events were classified as grade 2 (mild), including perihepatic hemorrhage and ipsilateral pneumothorax without clinical consequences, except one grade 3 pneumothorax requiring chest tube placement.

**Table 2:** Complication chart with management and outcomes

Case	Group	Adverse event	CIRSE Grade	Management	Outcome
1	CTHA	Pneumothorax	III	Thoracic drainage	No sequelae
2	CTHA	Hemoperitoneum	II	Conservative management	No sequelae
3	CTHA	Hemoperitoneum	II	Conservative management	No sequelae
4	CTHA	Pneumothorax	II	Conservative management	No sequelae
5	CTHA	Hemoperitoneum	II	Conservative management	No sequelae
6	CTHA	Arterioportal fistula	II	Conservative management	No sequelae
7	IOUS	Multiorgan failure	VI	Relaparotomy for intestinal perforation	Death
8	IOUS	Biliary fistula	III	Transcatheter biliary embolization	No sequelae
9	IOUS	Multiorgan failure	VI	Hemorrhagic shock after liver mobilization	Death
10	US	Hemoperitoneum	II	Conservative management	No sequelae
11	US	Arterioportal fistula	II	Conservative management	No sequelae
12	US	Arterioportal fistula	II	Conservative management	No sequelae
13	US	Biliary–colic fistula	III	Percutaneous abdominal drainage	No sequelae

Post-treatment, tumor progression in other liver segments occurred in 58/111 patients (52.3%), with 57 (51.3%) requiring further interventions, including repeat MWA, OLT, TACE, TARE or systemic therapy. One patient was eligible only for best supportive care. Median progression-free survival (PFS) was 11 months (range 1–90 months), distributed as follows: 9 months (range 1–41) for the US-guided group, 26 months (range 1–90) for the IOUS-guided group and 9 months (range 1–26) for the CTHA-guided group.

A total of 28 deaths occurred (25.2%), with notable variation between groups: 14/54 patients (25.9%) in the US group, 13/35 (37.1%) in the IOUS-guided group and 1/22 (4.5%) in the CTHA-guided group. Multivariate analysis showed significantly lower mortality risk for both CTHA-guided (*p* = 0.001) and US-guided (*p* = 0.049) MWAs compared to IOUS-guided procedures.

**Fig. 3:**
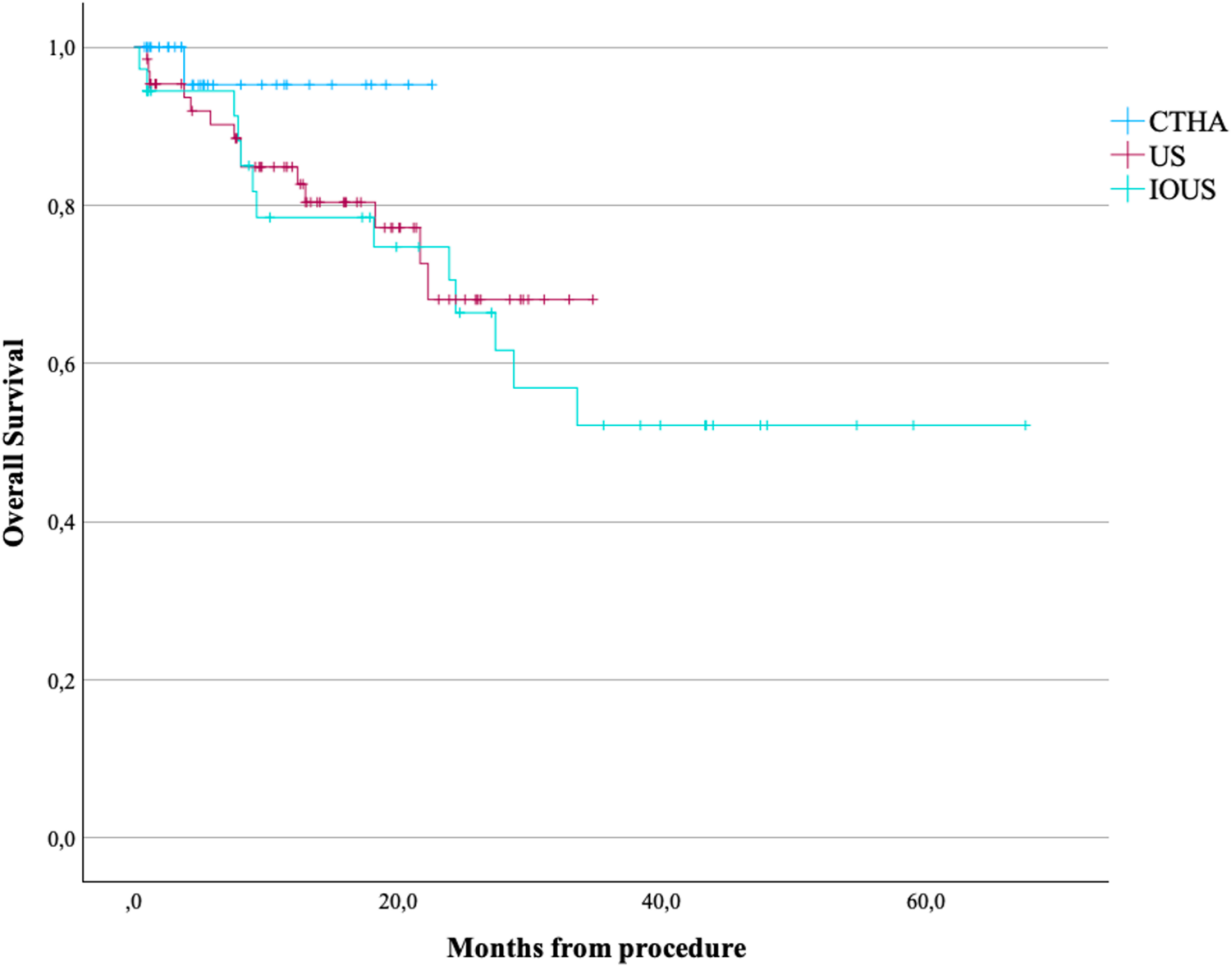
Kaplan–Meier curve describing the overall survival at 5 years of the three populations after the first MWA

Median overall survival (OS) for the entire cohort was 24 months (range 0–81). By subgroup, the median OS was 27 months (range 3–44) for the US-guided group, 34 months (range 0–81) for the IOUS-guided group and 15 months (range 6–26) for the CTHA-guided group (Fig. 3: Kaplan–Meier curve describing the overall survival at 5 years of the three populations after the first MWA). No statistically significant differences in OS (*p* = 0.07) or PFS (*p* = 0.584) were observed among the three guidance groups.

On univariate analysis (Table 3), the IOUS-guided approach was significantly associated with higher radicality (OR 3.31, 95%CI 1.15–11.98, *p* = 0.04), although this did not translate into improved survival (OR 0.89, 95%CI 0.32–2.32, *p* = 0.76). After a stepwise regression, at the multivariable analysis (Table 4) CTHA-guidance was associated with a twofold increase, though not statistically significant, in the odds of complete ablation compared to US-guidance (OR 2.03, 95% CI 0.73–6.28, *p* = 0.19), while IOUS-guidance showed a statistically significant threefold increase (OR 3.33, 95% CI 1.10–12.66, *p* = 0.04). Smaller tumor size was also a significant predictor of radicality, confirmed by both univariate (OR 0.93, 95% CI 0.89–0.98, *p* = 0.002) and multivariable analysis (OR 0.94, 95% CI 0.90–0.99, *p* = 0.01).

**Table 3:** Univariate analysis with logistic regressions on factors impacting on radicality

Factors	Radicality (%)	OR (95% CI)	*p*
*Group*			
US	72/89 (80.9)	1	
CTHA	45/51 (88.2)	1.77 (0.68–5.21)	0.2639
IOUS	56/60 (93.3)	3.31 (1.15–11.98)	0.0405
*Nodules*			
1	81/94 (86.2)	1	
2	19/25 (76)	0.38 (0.14–1.08)	0.0619
> 3	15/17 (88.2)	1.28 (0.21–24.83)	0.8206
*Adverse events*			
No	88/103 (85.4)	1	
Yes	8/13 (61.5)	0.29 (0.09–1.05)	*0.0481*
*A.E. classification*			
1–3	5/9 (55.6)	1	
4–6	3/4 (75)	2.4 (0.2–59.67)	0.5121
*Antenna repositioning*			
No	156/178 (87.6)	1	
Yes	17/22 (77.3)	0.48 (0.17–1.57)	0.1873

**Table 4:** Multivariate analysis on factors with *p* < 0.05 in the univariate analysis

Characteristics	OR (95% CI)	*p*
*Group*		
US	1	
CTHA	2.03 (0.73–6.28)	0.1912
IOUS	3.33 (1.10–12.66)	0.0485
*Dimensions*	0.94 (0.90–0.99)	0.0127

The risk of complications increased significantly with the number of nodules treated per session (OR 2.36, 95% CI 1.34–4.34, *p* = 0.003), which also led to prolonged hospital stays (OR 1.09, 95% CI 1.02–1.18, *p* = 0.01). Furthermore, adverse events were negatively associated with the likelihood of achieving radicality (OR 0.25, 95% CI 0.07–0.94, *p* = 0.032).

## Discussion

This study offers a comprehensive comparison of clinical and technical outcomes of MWA for HCC using three different imaging guidance modalities: US-guidance, IOUS-guidance and CTHA-guidance. The novelty of our study is to directly compare these three techniques in a single-center cohort, with particular emphasis on ablation radicality, complication rates and oncological outcomes.

The radicality rates observed across the US-guidance, IOUS-guidance and CTHA-guidance groups are broadly in line with what has been previously reported in the literature[26–28]. US-guided MWA remains the most established technique, backed by numerous studies demonstrating high rates of complete ablation, particularly in superficially located and well-visualized lesions[29]. IOUS-guided ablation, less frequently investigated, has shown promising results in selected surgical patients, with retrospective analyses indicating excellent radicality due to the advantages of intraoperative control[30, 31]. In contrast, literature on CTHA-guidance for HCC ablation is still limited, due to its prevalent use for treating colorectal liver metastases[32–35]. This study contributes to filling this gap by providing a structured clinical and technical assessment of this approach, obtaining a rate of oncological radicality of 88% without a corresponding increase in major complication rates and need for surgical assistance.

US-guided MWA remains an attractive and widely adopted technique for lesions that are clearly visualized on B-mode ultrasound[21]. The feasibility of performing the procedure under conscious sedation, coupled with short procedural times, supports its continued use[20]. Nevertheless, as corroborated by multiple studies, this approach is highly operator-dependent and less effective for isoechoic or deep-seated lesions, which can limit radicality rates and negatively influence long-term outcomes[36], as seen in our analysis. For the aforementioned drawbacks, the use of US-guidance might be limited to technically straightforward treatments, to avoid a meaningful increase in incomplete ablations of deep/inconspicuous HCCs, as well as procedure-related adverse events.

The observed superiority of IOUS-guidance in achieving complete ablation is consistent with literature, where IOUS has been shown to allow unparalleled accuracy in lesion localization and probe placement[37]. The real-time feedback and direct contact with the liver surface enable precise control of treatment margins. However, these advantages must be weighed against the inherent risks of a surgical approach, which is associated with higher morbidity and mortality [24]. As highlighted by previous surgical series, the complication rate in IOUS-guided interventions is not negligible, a finding also reflected in the two fatal events observed in our IOUS-guided MWA group[38]. So, in our view, operators should carefully balance the oncologic benefit associated with a radicality rate > 90% against the potential for adverse events, including fatal complications, when performing IOUS-guided interventions.

CTHA-guided MWA, although technically complex and resource-intensive, demonstrated excellent targeting capabilities. The combination of angiographic and CT imaging allows for detailed lesion visualization, particularly in cases where conventional imaging fails to clearly delineate the tumor [39, 40]. Previous reports have hinted at the potential of CTHA to improve lesion conspicuity and procedural planning [10, 14, 41]], although data remain sparse for HCC [[10, 14, 39–41]. Today, in fact, CTHA is widely used for guiding MWAs of colorectal liver metastases, and our aim was to translate this experience to a completely different disease entity, namely HCC in a cirrhotic subset. In our experience, the initially higher radiation doses associated with CTHA were primarily observed during the early phase of adoption, likely due to the learning curve and the need for multiple scans. Over time, as procedural efficiency improved, a marked reduction in radiation exposure was noted, aligning with safety profiles reported in routine CT-guided MWAs protocols[42]: in the very first patient, presenting with four HCC nodules, the delivered radiation dose was notably high (13.797 mGy cm^2^), whereas in the most recent one, affected by a single nodule, it was markedly lower (983 mGy cm^2^).

PFS rates in our cohort closely reflect those found in existing studies, further confirming the efficacy of MWA in early-stage HCC across different guidance modalities[43]. While OS appeared slightly lower than expected in the CTHA-guided MWA cohort, it is important to interpret this finding cautiously. Literature on OS in MWA-treated HCC patients shows a wide variability, often influenced by patient selection, comorbidities and follow-up duration[44, 45]. In our study, the shorter follow-up period in this subgroup might have contributed to an underestimation of late events, thus limiting direct comparability with the other two groups.

A point worth discussing is the observed trend in mortality distribution among the three groups. Although the deaths were more frequent in the IOUS and US-guided group, this difference did not translate into statistically significant disparities in PFS or OS. A possible explanation for this discrepancy lies in the differing follow-up durations; however, it is intuitive how IOUS-guided procedures carried a higher mortality rate, inherently related to their classification as major surgical interventions[23, 46].

Interestingly, in our analysis, radicality was not statistically associated with improved OS. This finding may reflect the unique prognostic landscape of cirrhotic patients, where underlying liver function and comorbidities often outweigh tumor-specific variables in determining long-term outcomes[47]. Particularly in our cohort, patients who underwent MWA as part of a bridge-to-transplant strategy exhibited excellent survival, with no HCC-related deaths recorded, confirming previous results[48]. In these cases, mortality was exclusively related to complications arising from liver transplantation itself, further supporting the concept that in cirrhotic patients, survival is more tightly linked to hepatic function and transplant-related factors than to the local eradication of HCC alone[49].

Importantly, our study highlights three guidance techniques commonly used in clinical practice. However, it should be noted that other methods—such as conventional CT-guidance, fusion imaging, augmented reality or robotic-assistance—have also been described in the literature, each with specific advantages and limitations[50–54]. Comparative analyses between these modalities remain limited, and further research is necessary to define their role in clinical algorithms, particularly in complex or borderline cases [55].

An additional point concerns the different resources required by the three guidance techniques. Percutaneous US-guidance is typically the least resource-intensive, relying on standard US equipment and moderate sedation, with procedure times often < 60 min [56]. In contrast, IOUS-guidance requires a fully equipped operating room, at least two operators, and general anesthesia, with longer operative times, particularly in patients with extensive abdominal adhesions [24]. CTHA-guided procedures require a costly hybrid angio suite or a CT suite with a mobile C-arm and a longer setup, due to the both endovascular and percutaneous phases, which may limit feasibility in high-workflow settings [40]. In our opinion, all these factors should be weighed alongside efficacy and safety, when selecting the preferred approach.

Several limitations of our analysis should be acknowledged. First, the retrospective nature of this study may have introduced selection bias, despite the use of a prospectively maintained institutional database ensuring completeness and accuracy of clinical data. Second, the technical performance of both IOUS- and CTHA-guided procedures was potentially affected by the learning curve, as all procedures from the initial experience were included. Moreover, in relation to our institutional workflow, missing a randomization between the image-guidance techniques possibly biased some of the observed outcomes, particularly the radicality and complication rates, and the differences in follow-up could have hindered the PFS and OS outcomes.

## Conclusions

In this single-center experience, IOUS-guided MWA for HCC was associated with the highest rate of radical ablation, reflecting the superior visualization and control afforded by intraoperative imaging. However, this advantage came at the cost of increased severe complication rates, likely due to the surgical nature of the procedure. Conversely, CTHA-guided ablations demonstrated an excellent balance of safety and efficacy, with high radicality and minimal severe adverse events, albeit requiring specialized expertise. US-guided MWA, while safe and fast for favorable cases, showed a lower radicality rate and was highly dependent on operator skill and lesion visibility.

Although retrospective and non-randomized, these findings support a tailored approach to imaging guidance in MWA, considering lesion characteristics, patient condition and institutional resources to optimize outcomes.

## References

[1] Qiu S, Cai J, Yang Z et al (2024) Trends in hepatocellular carcinoma mortality rates in the US and projections through 2040. JAMA Netw Open 7:e2445525. 10.1001/jamanetworkopen.2024.45525 39556395 10.1001/jamanetworkopen.2024.45525 PMC11574689

[2] Facciorusso A, Di Maso M, Muscatiello N (2016) Microwave ablation versus radiofrequency ablation for the treatment of hepatocellular carcinoma: a systematic review and meta-analysis. Int J Hyperthermia 32:339–344. 10.3109/02656736.2015.1127434 26794414 10.3109/02656736.2015.1127434

[3] Lee P, Makkena A, Tantawi M et al (2023) Microwave ablation as a primary versus secondary treatment for hepatocellular carcinoma. Diagn Interv Radiol 29:359–366. 10.4274/dir.2023.221930 36988024 10.4274/dir.2023.221930 PMC10679698

[4] Lanza C, Angileri SA, Biondetti P et al (2024) Percutaneous microwave ablation of HCC: comparison between 100 and 150 W technology systems. Radiol Med 129:1916–1925. 10.1007/s11547-024-01927-3 39514155 10.1007/s11547-024-01927-3

[5] Liu K, Zheng H, Sui X et al (2023) Microwave ablation versus surgical resection for subcapsular hepatocellular carcinoma: a propensity score-matched study of long-term therapeutic outcomes. Eur Radiol 33:1938–1948. 10.1007/s00330-022-09135-1 36114849 10.1007/s00330-022-09135-1

[6] Gaia S, Ciruolo M, Ribaldone DG et al (2021) Higher efficiency of percutaneous microwave (MWA) than radiofrequency ablation (RFA) in achieving complete response in cirrhotic patients with early hepatocellular carcinoma. Curr Oncol 28:1034–1044. 10.3390/curroncol28020101 33669107 10.3390/curroncol28020101 PMC8025753

[7] Minami Y, Aoki T, Hagiwara S, Kudo M (2023) Tips for preparing and practicing thermal ablation therapy of hepatocellular carcinoma. Cancers (Basel). 10.3390/cancers15194763 37835456 10.3390/cancers15194763 PMC10571938

[8] Jin T, Liu X, Zhang H et al (2020) Ultrasound-guided percutaneous microwave ablation for hepatocellular carcinoma adjacent to large vessels: a propensity score matching analysis. Int J Hyperthermia 37:955–964. 10.1080/02656736.2020.1804076 32781862 10.1080/02656736.2020.1804076

[9] Cillo U, Bertacco A, Fasolo E et al (2019) Videolaparoscopic microwave ablation in patients with HCC at a European high-volume center: results of 815 procedures. J Surg Oncol 120:956–965. 10.1002/jso.25651 31373009 10.1002/jso.25651

[10] Puijk RS, Dijkstra M, van der Lei S et al (2024) The added value of transcatheter CT hepatic angiography (CTHA) image guidance in percutaneous thermal liver ablation: an experts’ opinion pictorial essay. Cancers (Basel). 10.3390/cancers16061193 38539527 10.3390/cancers16061193 PMC10969731

[11] Tsoumakidou G, Saltiel S, Villard N et al (2021) Image-guided marking techniques in interventional radiology: a review of current evidence. Diagn Interv Imaging 102:699–707. 10.1016/j.diii.2021.07.002 34419388 10.1016/j.diii.2021.07.002

[12] Minami Y, Kudo M (2021) Image guidance in ablation for hepatocellular carcinoma: contrast-enhanced ultrasound and fusion imaging. Front Oncol 11:593636. 10.3389/fonc.2021.593636 33747913 10.3389/fonc.2021.593636 PMC7973273

[13] Cillo U, Vitale A, Dupuis D et al (2013) Laparoscopic ablation of hepatocellular carcinoma in cirrhotic patients unsuitable for liver resection or percutaneous treatment: a cohort study. PLoS ONE 8:e57249. 10.1371/journal.pone.0057249 23437351 10.1371/journal.pone.0057249 PMC3578795

[14] van der Lei S, Opperman J, Dijkstra M et al (2023) The added diagnostic value of transcatheter CT hepatic arteriography for intraprocedural detection of previously unknown colorectal liver metastases during percutaneous ablation and impact on the definitive treatment plan. Cardiovasc Intervent Radiol 46:1257–1266. 10.1007/s00270-023-03508-9 37491521 10.1007/s00270-023-03508-9 PMC10471708

[15] Reig M, Forner A, Rimola J et al (2022) BCLC strategy for prognosis prediction and treatment recommendation: the 2022 update. J Hepatol 76:681–693. 10.1016/j.jhep.2021.11.018 34801630 10.1016/j.jhep.2021.11.018 PMC8866082

[16] Chernyak V, Fowler KJ, Kamaya A et al (2018) Liver Imaging Reporting and Data System (LI-RADS) version 2018: imaging of hepatocellular carcinoma in at-risk patients. Radiology 289:816–830. 10.1148/radiol.2018181494 30251931 10.1148/radiol.2018181494 PMC6677371

[17] Filippiadis DK, Binkert C, Pellerin O et al (2017) CIRSE quality assurance document and standards for classification of complications: the CIRSE classification system. Cardiovasc Intervent Radiol 40:1141–1146. 10.1007/s00270-017-1703-4 28584945 10.1007/s00270-017-1703-4

[18] Puijk RS, Ahmed M, Adam A et al (2021) Consensus guidelines for the definition of time-to-event end points in image-guided tumor ablation: results of the SIO and DATECAN initiative. Radiology 301:533–540. 10.1148/radiol.2021203715 34581627 10.1148/radiol.2021203715

[19] Hendriks P, Boel F, Oosterveer TT et al (2023) Ablation margin quantification after thermal ablation of malignant liver tumors: how to optimize the procedure? A systematic review of the available evidence. Eur J Radiol Open 11:100501. 10.1016/j.ejro.2023.100501 37405153 10.1016/j.ejro.2023.100501 PMC10316004

[20] Jung CFM, Liverani E, Binda C et al (2024) Non-operating room anesthesia (NORA) for ultrasound-guided liver radiofrequency ablation. Diagnostics. 10.3390/diagnostics14161783 39202272 10.3390/diagnostics14161783 PMC11353362

[21] Kim JW, Shin SS, Heo SH et al (2015) Ultrasound-guided percutaneous radiofrequency ablation of liver tumors: how we do it safely and completely. Korean J Radiol 16:1226–1239. 10.3348/kjr.2015.16.6.1226 26576111 10.3348/kjr.2015.16.6.1226 PMC4644743

[22] Gruttadauria S, Pagano D, Tropea A et al (2016) Laparoscopic approach for thermoablation microwave in the treatment of hepatocellular carcinoma: a single center experience. J Laparoendosc Adv Surg Tech A 26:808–811. 10.1089/lap.2016.0373 27508328 10.1089/lap.2016.0373

[23] Wang T, Zhang X-Y, Lu X, Zhai B (2019) Laparoscopic microwave ablation of hepatocellular carcinoma at liver surface: technique effectiveness and long-term outcomes. Technol Cancer Res Treat 18:1533033818824338. 10.1177/1533033818824338 30803390 10.1177/1533033818824338 PMC6378635

[24] Muglia R, Marra P, Pinelli D et al (2023) Technical and clinical outcomes of laparoscopic-laparotomic hepatocellular carcinoma thermal ablation with microwave technology: case series and review of literature. Cancers (Basel). 10.3390/cancers16010092 38201536 10.3390/cancers16010092 PMC10778313

[25] Smits MLJ, Bruijnen RCG, Tetteroo P et al (2023) Hepatic arteriography and C-Arm CT-guided ablation (HepACAGA) to improve tumor visualization, navigation and margin confirmation in percutaneous liver tumor ablation. Cardiovasc Intervent Radiol 46:1365–1374. 10.1007/s00270-023-03545-4 37704863 10.1007/s00270-023-03545-4 PMC10547639

[26] Dong T-T, Wang L, Li M et al (2023) Clinical results, risk factors, and future directions of ultrasound-guided percutaneous microwave ablation for hepatocellular carcinoma. J Hepatocell Carcinoma 10:733–743. 10.2147/JHC.S409011 37215363 10.2147/JHC.S409011 PMC10198179

[27] Vouche M (2023) Large-scale data from real-life practice of percutaneous liver thermal ablation from an international registry: unconditionally trustful Atlas or colossus with feet of clay? Eur Radiol 34:3320–3321. 10.1007/s00330-023-10519-0 38112767 10.1007/s00330-023-10519-0

[28] Ierardi AM, Giorlando F, Piacentino F et al (2017) Factors predicting outcomes of microwave ablation of small hepatocellular carcinoma. Radiol Med 122:81–87. 10.1007/s11547-016-0694-6 27744607 10.1007/s11547-016-0694-6

[29] Zhang T-T, Luo H-C, Cui X et al (2015) Ultrasound-guided percutaneous microwave ablation treatment of initial recurrent hepatocellular carcinoma after hepatic resection: long-term outcomes. Ultrasound Med Biol 41:2391–2399. 10.1016/j.ultrasmedbio.2015.04.019 26074453 10.1016/j.ultrasmedbio.2015.04.019

[30] Baker EH, Thompson K, McKillop IH et al (2017) Operative microwave ablation for hepatocellular carcinoma: a single center retrospective review of 219 patients. J Gastrointest Oncol 8:337–346. 10.21037/jgo.2016.09.06 28480072 10.21037/jgo.2016.09.06 PMC5401852

[31] Della Corte A, Ratti F, Monfardini L et al (2020) Comparison between percutaneous and laparoscopic microwave ablation of hepatocellular carcinoma. Int J Hyperthermia 37:542–548. 10.1080/02656736.2020.1769869 32469252 10.1080/02656736.2020.1769869

[32] Puijk RS, Nieuwenhuizen S, van den Bemd BAT et al (2020) Transcatheter CT hepatic arteriography compared with conventional CT fluoroscopy guidance in percutaneous thermal ablation to treat colorectal liver metastases: a single-center comparative analysis of 2 historical cohorts. J Vasc Interv Radiol 31:1772–1783. 10.1016/j.jvir.2020.05.011 32981819 10.1016/j.jvir.2020.05.011

[33] van der Lei S, Puijk RS, Dijkstra M et al (2025) Thermal ablation versus surgical resection of small-size colorectal liver metastases (COLLISION): an international, randomised, controlled, phase 3 non-inferiority trial. Lancet Oncol 26:187–199. 10.1016/S1470-2045(24)00660-0 39848272 10.1016/S1470-2045(24)00660-0

[34] Paolucci I, Lin Y-M, Jones AK et al (2023) Use of contrast media during CT-guided thermal ablation of colorectal liver metastasis for procedure planning is associated with improved immediate outcomes. Cardiovasc Intervent Radiol 46:327–336. 10.1007/s00270-022-03333-6 36609863 10.1007/s00270-022-03333-6 PMC10446157

[35] Schembri V, Piron L, Le Roy J et al (2020) Percutaneous ablation of obscure hypovascular liver tumours in challenging locations using arterial CT-portography guidance. Diagn Interv Imaging 101:707–713. 10.1016/j.diii.2020.09.005 33012694 10.1016/j.diii.2020.09.005

[36] Fu Y, Zhu Q, Zhao X et al (2025) Efficacy and safety of ultrasound-guided percutaneous microwave ablation for hepatocellular carcinoma at specific anatomic sites of the liver: a systematic review and meta-analysis. BMC Gastroenterol 25:505. 10.1186/s12876-025-04081-w 40624629 10.1186/s12876-025-04081-w PMC12232681

[37] Santambrogio R, Vertemati M, Barabino M, Zappa MA (2023) Laparoscopic microwave ablation: which technologies improve the results. Cancers (Basel). 10.3390/cancers15061814 36980701 10.3390/cancers15061814 PMC10046461

[38] Cillo U, Noaro G, Vitale A et al (2014) Laparoscopic microwave ablation in patients with hepatocellular carcinoma: a prospective cohort study. HPB (Oxford) 16:979–986. 10.1111/hpb.12264 24750429 10.1111/hpb.12264 PMC4487748

[39] Li L, Liu L-Z, Xie Z-M et al (2004) Multi-phasic CT arterial portography and CT hepatic arteriography improving the accuracy of liver cancer detection. World J Gastroenterol 10:3118–3121. 10.3748/wjg.v10.i21.3118 15457555 10.3748/wjg.v10.i21.3118 PMC4611253

[40] Muglia R, Gargiulo C, Carbone FS et al (2025) CT hepatic arteriography for improved detection and ablation of occult HCC nodules: a retrospective analysis. Cardiovasc Intervent Radiol. 10.1007/s00270-025-04276-4 41266805 10.1007/s00270-025-04276-4

[41] Albuquerque J, Lin Y-M, Paolucci I et al (2024) Incidental ring-hyperenhancing liver micronodules at CT hepatic arteriography-guided percutaneous thermal ablation of colorectal liver metastases. Radiol Imaging Cancer 6:e230099. 10.1148/rycan.230099 38363196 10.1148/rycan.230099 PMC10988328

[42] McCarthy CJ, Kilcoyne A, Li X et al (2018) Radiation dose and risk estimates of CT-guided percutaneous liver ablations and factors associated with dose reduction. Cardiovasc Intervent Radiol 41:1935–1942. 10.1007/s00270-018-2066-1 30132100 10.1007/s00270-018-2066-1

[43] Hermida M, Cassinotto C, Piron L et al (2020) Multimodal percutaneous thermal ablation of small hepatocellular carcinoma: predictive factors of recurrence and survival in western patients. Cancers (Basel) 12:e0313. 10.3390/cancers12020313 10.3390/cancers12020313 PMC7072144 32013112

[44] Ma S, Ding M, Li J et al (2017) Ultrasound-guided percutaneous microwave ablation for hepatocellular carcinoma: clinical outcomes and prognostic factors. J Cancer Res Clin Oncol 143:131–142. 10.1007/s00432-016-2266-5 27650934 10.1007/s00432-016-2266-5 PMC11819308

[45] Serbanescu-Kele Apor CMC, Ruiter SJS, van den Berg AP et al (2022) Outcomes after primary and repeat thermal ablation of hepatocellular carcinoma with or without liver transplantation. Eur Radiol 32:4168–4176. 10.1007/s00330-021-08515-3 35133486 10.1007/s00330-021-08515-3 PMC9123025

[46] Jiang B, Yan X-F, Zhang J-H (2018) Meta-analysis of laparoscopic versus open liver resection for hepatocellular carcinoma. Hepatol Res 48:635–663. 10.1111/hepr.13061 29330919 10.1111/hepr.13061

[47] Liu Y-B, Chen M-K (2022) Epidemiology of liver cirrhosis and associated complications: current knowledge and future directions. World J Gastroenterol 28:5910–5930. 10.3748/wjg.v28.i41.5910 36405106 10.3748/wjg.v28.i41.5910 PMC9669831

[48] Couillard AB, Knott EA, Zlevor AM et al (2022) Microwave ablation as bridging to liver transplant for patients with hepatocellular carcinoma: a single-center retrospective analysis. J Vasc Interv Radiol 33:1045–1053. 10.1016/j.jvir.2022.05.019 35667580 10.1016/j.jvir.2022.05.019

[49] Al-Smadi K, Qureshi A, Buitrago M et al (2024) Survival and disease progression in older adult patients with cirrhosis: a retrospective study. Int J Hepatol 2024:5852680. 10.1155/2024/5852680 39149542 10.1155/2024/5852680 PMC11326880

[50] Yin T, Li W, Zhao P et al (2017) Treatment efficacy of CT-guided percutaneous microwave ablation for primary hepatocellular carcinoma. Clin Radiol 72:136–140. 10.1016/j.crad.2016.10.022 27890422 10.1016/j.crad.2016.10.022

[51] Yang J, Liang S, Liu H et al (2023) Efficacy and safety of microwave ablation assisted by ultrasound fusion imaging for primary and secondary liver cancers with a diameter of 3–7 cm. J Hepatocell Carcinoma 10:1839–1848. 10.2147/JHC.S424009 37873028 10.2147/JHC.S424009 PMC10590584

[52] Solbiati M, Ierace T, Muglia R et al (2022) Thermal ablation of liver tumors guided by augmented reality: an initial clinical experience. Cancers (Basel). 10.3390/cancers14051312 35267620 10.3390/cancers14051312 PMC8909771

[53] Calandri M, Ruggeri V, Carucci P et al (2019) Thermal ablation with fusion imaging guidance of hepatocellular carcinoma without conspicuity on conventional or contrast-enhanced US: surrounding anatomical landmarks matter. Radiol Med 124:1043–1048. 10.1007/s11547-019-01057-1 31270723 10.1007/s11547-019-01057-1

[54] Milot L, L’Huillier R, Dumortier J et al (2023) Robotic-assisted percutaneous microwave ablation of hepatocellular carcinoma. Diagn Interv Imaging 104:258–260. 10.1016/j.diii.2023.01.011 36792426 10.1016/j.diii.2023.01.011

[55] Puijk RS, Dijkstra M, van den Bemd BAT et al (2022) Improved outcomes of thermal ablation for colorectal liver metastases: a 10-year analysis from the prospective Amsterdam CORE registry (AmCORE). Cardiovasc Intervent Radiol 45:1074–1089. 10.1007/s00270-022-03152-9 35585138 10.1007/s00270-022-03152-9 PMC9307533

[56] Crocetti L, de Baére T, Pereira PL, Tarantino FP (2020) CIRSE standards of practice on thermal ablation of liver tumours. Cardiovasc Intervent Radiol 43:951–962. 10.1007/s00270-020-02471-z 32382856 10.1007/s00270-020-02471-z

